# Association Between Caffeine Citrate Initiation Within the First 2 h of Life and Respiratory Outcomes in Very Preterm Infants: A Single-Center Observational Cohort Study

**DOI:** 10.3390/children13070968

**Published:** 2026-07-22

**Authors:** Halil Ugur Hatipoglu, Birgul Livaoglu Say, Nurdan Uras

**Affiliations:** 1 Department of Pediatrics, University of Health Sciences, Haseki Training and Research Hospital, Istanbul 34270, Türkiye; 2 Department of Pediatrics, Division of Neonatology, Alanya Alaaddin Keykubat University, Antalya 07425, Türkiye; 3 Department of Pediatrics, Division of Neonatology, Istinye University, Bahçeşehir Liv Hospital, Istanbul 34517, Türkiye

**Keywords:** bronchopulmonary dysplasia, caffeine citrate, neonatal intensive care, preterm infant, respiratory support

## Abstract

**Highlights:**

**What are the main findings?**

• Caffeine citrate initiation after the first 2 h of life was associated with higher odds of bronchopulmonary dysplasia and the composite outcome of bronchopulmonary dysplasia or death in very preterm infants.
• These associations persisted in expanded stabilized inverse probability of treatment weighting analyses incorporating maternal, placental, perinatal, and golden-hour respiratory characteristics, although the estimates remained imprecise.

**What are the implications of the main findings?**

• Caffeine initiation timing may represent a clinically relevant component of early neonatal care, but it may also reflect differences in early illness severity and stabilization requirements.
• Prospective multicenter studies are needed to determine whether protocolized very early caffeine initiation independently improves respiratory outcomes.

**Abstract:**

**Background/Objectives:** Caffeine citrate is widely used in very preterm infants, but whether initiation during the first postnatal hours is independently associated with respiratory outcomes remains uncertain. We evaluated the association between caffeine initiation within versus after the first 2 h of life and respiratory outcomes in infants born at <32 weeks’ gestation. **Methods:** This single-center observational cohort study included 84 infants born at <32 weeks’ gestation and with a birth weight of ≤1500 g who received caffeine citrate within the first 24 h of life. The 2 h threshold represented the unit’s protocol-defined target for caffeine loading rather than a biologically validated cutoff. The primary outcome was bronchopulmonary dysplasia (BPD) at 36 weeks’ postmenstrual age. Secondary outcomes included BPD or death, moderate/severe BPD or death, respiratory support requirements, and neonatal morbidities. Conventional multivariable logistic regression and expanded gestational age- and birth weight-based propensity score models with stabilized inverse probability of treatment weighting (IPTW) were used. The expanded propensity score models incorporated maternal, placental, perinatal, and early respiratory variables, including first-hour invasive mechanical ventilation and surfactant administration within the first hour. **Results:** Caffeine was initiated within the first 2 h in 41 infants and after the first 2 h in 43 infants. BPD occurred in 10/41 (24.4%) and 24/40 (60.0%) infants, respectively, while BPD or death occurred in 10/41 (24.4%) and 27/43 (62.8%), respectively. In expanded IPTW analyses, caffeine initiation after the first 2 h remained associated with BPD in both the gestational age-based model (OR 3.40, 95% CI 1.22–9.48) and the birth weight-based model (OR 3.23, 95% CI 1.16–9.00). Corresponding ORs for BPD or death were 3.89 (95% CI 1.41–10.71) and 3.68 (95% CI 1.34–10.12). Additional adjustment for residual imbalance in pretreatment respiratory support produced similar estimates. **Conclusions:** Caffeine initiation after the first 2 h of life was associated with higher odds of BPD and BPD or death. These findings support further investigation of very early caffeine timing but do not establish a causal effect because of the observational design, modest sample size, and potential residual confounding.

## 1. Introduction

Bronchopulmonary dysplasia (BPD) remains a major respiratory morbidity among very preterm infants. Although earlier definitions were largely based on oxygen requirement, radiographic findings, and chronic lung injury following neonatal respiratory failure, the phenotype of BPD has evolved with advances in neonatal care, including antenatal corticosteroids, surfactant therapy, noninvasive ventilation, and improved survival of extremely preterm infants [1]. Contemporary severity classifications increasingly emphasize the level of respiratory support required at 36 weeks’ postmenstrual age (PMA), which more directly reflects functional respiratory disease burden in very preterm infants [2].

Caffeine citrate is one of the most frequently used medications in neonatal intensive care and is widely accepted as the preferred methylxanthine for apnea of prematurity [3]. The Caffeine for Apnea of Prematurity trial demonstrated that caffeine treatment reduced the incidence of BPD and improved survival without neurodevelopmental disability at 18 to 21 months of corrected age in very low birth weight infants [3,4]. Subsequent observational studies and meta-analyses have suggested that earlier caffeine initiation may be associated with shorter respiratory support, less exposure to mechanical ventilation, and a lower incidence of BPD [5,6,7,8].

However, “early” caffeine has been defined inconsistently, most commonly as administration within the first 24–72 h of life [6,7,8]. Fewer studies have evaluated caffeine administration during delivery-room stabilization or the first postnatal hours, and most have been feasibility, pilot, or relatively small randomized studies that were not powered for outcomes such as BPD [9,10,11,12].

The first postnatal hours are also a period of substantial clinical heterogeneity. Caffeine timing may be influenced by the need for respiratory stabilization, intubation, surfactant administration, vascular access, and other urgent interventions. Consequently, delayed administration may function partly as a marker of early illness severity or unit workflow rather than as an isolated exposure. Maternal, placental, and perinatal complications may also influence both early stabilization requirements and subsequent neonatal outcomes [13]. Analyses of very early caffeine timing therefore require detailed adjustment for these potential sources of confounding.

This study evaluated the association between caffeine citrate initiation within versus after the first 2 h of life and respiratory outcomes, including BPD and BPD or death, in infants born at <32 weeks’ gestation. The 2 h threshold reflected the unit’s protocol-defined target for early caffeine loading and should be interpreted as an operational clinical cutoff rather than a biological or pharmacodynamic threshold. We hypothesized that initiation after the first 2 h would be associated with less favorable respiratory outcomes. Conventional multivariable analyses and expanded propensity score-based analyses incorporating maternal, placental, perinatal, and golden-hour respiratory variables were performed to assess the robustness of this association.

## 2. Materials and Methods

### 2.1. Study Design and Population

This single-center observational cohort study was conducted in the neonatal intensive care unit of Bahçeşehir Liv Hospital, a tertiary neonatal care center. All eligible infants born at <32 weeks’ gestation and with a birth weight of ≤1500 g who received caffeine citrate within the first 24 h of life between 1 August 2021 and 31 December 2022 were included.

Infants were classified according to whether caffeine citrate was initiated within the first 2 h of life or after the first 2 h but within the first 24 h. Exclusion criteria were gestational age ≥ 32 weeks and/or birth weight > 1500 g, major congenital anomalies, absence of parental consent, death within the first 24 h of life, incomplete or uncertain data on caffeine initiation timing, and transfer from another center after birth.

According to the unit protocol, caffeine loading was intended to be completed within the first 2 postnatal hours in eligible infants. This threshold was selected because it represented the unit’s routine target for completing initial stabilization and caffeine administration; it was not based on a previously validated biological or pharmacodynamic cutoff. In some infants, caffeine initiation was delayed beyond 2 h because respiratory stabilization, intubation, surfactant administration, umbilical venous catheter placement, or other urgent intensive care procedures took priority.

Infants who died before 36 weeks’ PMA and therefore could not be assessed for BPD were excluded from the survivor-based BPD outcome. They were retained in the composite outcomes of BPD or death and moderate/severe BPD or death.

All infants who met the eligibility criteria and for whom parental consent was obtained during the prespecified study period were included. No a priori sample size calculation was performed; therefore, the cohort size was determined by the number of eligible infants available during the study period.

**Figure 1:**
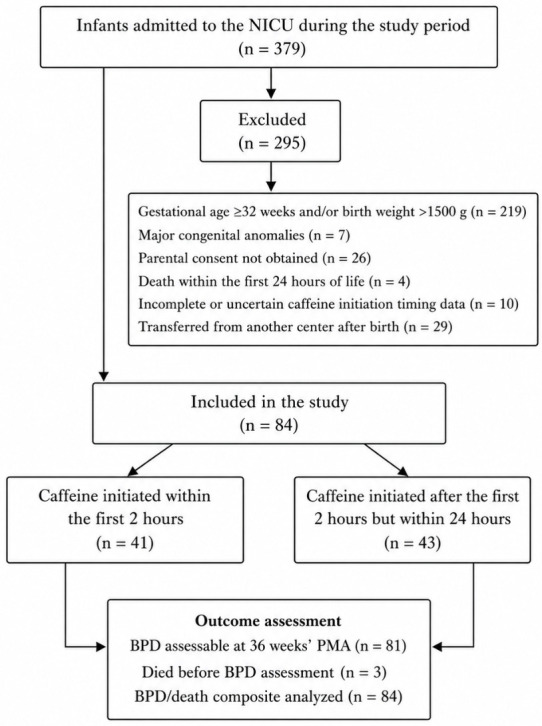
Flow diagram of participant selection. A total of 379 infants were admitted to the neonatal intensive care unit during the study period. After exclusion of infants who did not meet the gestational age or birth weight criteria, had major congenital anomalies, lacked parental consent, died within the first 24 h, had incomplete caffeine initiation timing data, or were transferred from another center after birth, 84 infants were included.

During the study period, 379 infants were admitted to the neonatal intensive care unit. A total of 295 infants were excluded: 219 because they were born at ≥32 weeks’ gestation and/or had a birth weight > 1500 g, seven because of major congenital anomalies, 26 because parental consent was not obtained, four because they died within the first 24 h of life, 10 because caffeine initiation timing data were incomplete or uncertain, and 29 because they were transferred from another center after birth. The final study cohort comprised 84 infants. Participant selection is summarized in Figure 1.

For contextual comparison, the unit-level incidence of BPD during the study period was calculated as the proportion of infants born at <32 weeks’ gestation with a birth weight of ≤1500 g who were assessable for BPD at 36 weeks’ PMA.

### 2.2. Caffeine Citrate Administration

Caffeine citrate was administered using the unit’s routine dosing protocol, consistent with commonly used neonatal dosing regimens, consisting of a 20 mg/kg loading dose followed by 10 mg/kg/day maintenance therapy, expressed as caffeine citrate [3,14].

The first maintenance dose was administered 24 h after the loading dose, and subsequent maintenance doses were administered at 24 h intervals.

Treatment was initially administered intravenously and was converted to oral administration after full enteral feeding had been achieved. The maintenance dose was adjusted according to body weight and clinical response.

Caffeine treatment duration was calculated from the initial loading dose to the final caffeine dose, and PMA at discontinuation was defined as PMA on the date of the final dose. In routine practice, caffeine was generally discontinued at approximately 33–34 weeks’ PMA after the infant had remained free of clinically significant apnea, bradycardia, or desaturation events requiring intervention for at least 5–7 days and no longer required positive-pressure respiratory support [15]. The final decision was individualized according to the attending neonatologist’s clinical assessment.

### 2.3. Outcomes

The primary outcome was BPD at 36 weeks’ PMA among infants who survived and were clinically assessable at that time. BPD severity was classified using the respiratory support-based definition proposed by Jensen et al. [2]. Infants requiring no respiratory support at 36 weeks’ PMA were classified as having no BPD. Grade 1 BPD was defined as nasal cannula flow ≤ 2 L/min, grade 2 BPD as nasal cannula flow > 2 L/min or noninvasive positive airway pressure, and grade 3 BPD as invasive mechanical ventilation. For clinical presentation, these categories were described as mild, moderate, and severe BPD, respectively.

Secondary respiratory outcomes included supplemental oxygen requirement at postnatal day 28 and supplemental oxygen requirement at 32, 34, and 36 weeks’ PMA; respiratory support requirement at the same time points; BPD severity; moderate/severe BPD; BPD or death; and moderate/severe BPD or death. Supplemental oxygen requirement was defined as an FiO > 21% at the corresponding assessment.

The composite outcome of BPD or death was defined as BPD at 36 weeks’ PMA or death before hospital discharge. The composite outcome of moderate/severe BPD or death was defined as grade 2 or grade 3 BPD at 36 weeks’ PMA or death before hospital discharge. Infants who fulfilled both components were counted only once. Infants who died before 36 weeks’ PMA were excluded from the BPD-only analyses but were retained in the composite outcome analyses.

Secondary neonatal outcomes included death, necrotizing enterocolitis stage ≥ II, retinopathy of prematurity stage 3–4, any intraventricular hemorrhage, intraventricular hemorrhage grade 3–4, pulmonary interstitial emphysema, early-onset neonatal sepsis, late-onset neonatal sepsis, and patent ductus arteriosus treatment.

Patent ductus arteriosus requiring treatment was defined as an echocardiographically confirmed PDA considered hemodynamically significant by the attending neonatologist and pediatric cardiologist and treated pharmacologically with ibuprofen or paracetamol and/or by surgical ligation.

Early-onset neonatal sepsis was defined as clinically diagnosed or culture-proven sepsis occurring within the first 72 h of life. Late-onset neonatal sepsis was defined as clinically diagnosed or culture-proven sepsis occurring after 72 h of life during the NICU hospitalization. Clinical sepsis was defined according to the contemporaneous diagnosis documented by the treating neonatology team, based on compatible clinical and laboratory findings and antimicrobial treatment.

Intraventricular hemorrhage was classified according to Papile et al. [16], necrotizing enterocolitis according to the modified Bell criteria [17], and retinopathy of prematurity according to the International Classification of Retinopathy of Prematurity [18].

### 2.4. Covariates and Early Respiratory Characteristics

Baseline maternal, placental, perinatal, and neonatal variables included gestational age, birth weight, Fenton-based small-for-gestational-age status, sex, maternal age, mode of delivery, multiple gestation, gestational diabetes mellitus, preeclampsia, premature or preterm premature rupture of membranes, placental abruption, cervical insufficiency, antenatal corticosteroid therapy, Apgar scores, and SNAPPE-II score when available.

Information on the indication for cesarean delivery and category-1 emergency cesarean delivery was not consistently available in the study dataset and therefore could not be incorporated into the adjusted analyses.

Early respiratory characteristics included the highest FiO_2_ requirement before caffeine administration, the highest respiratory support level before caffeine administration, receipt of any pretreatment respiratory support, invasive mechanical ventilation during the first hour of life, surfactant administration, and surfactant administration within the first hour of life.

Pretreatment respiratory support was categorized as absent, incubator oxygen, nasal continuous positive airway pressure, nasal synchronized intermittent mandatory ventilation, or invasive mechanical ventilation. “Any pretreatment respiratory support” indicated receipt of any of these forms of respiratory assistance before caffeine initiation.

Surfactant exposure was described using two complementary approaches. Individual surfactant treatment episodes were categorized according to clinical strategy as prophylactic, early rescue, or late selective administration. Prophylactic surfactant was defined as administration immediately after birth, before established clinical signs of respiratory distress syndrome. Early rescue surfactant was defined as administration after the development of respiratory distress syndrome within the first 2 h of life, whereas late selective surfactant was defined as administration after the first 2 h for persistent or worsening respiratory distress syndrome. Because some infants received more than one surfactant dose, these treatment-related variables were not mutually exclusive. Early surfactant therapy, as used in the conventional multivariable models, was defined as receipt of prophylactic and/or early rescue surfactant. To address potential confounding related to golden-hour respiratory management, infants were additionally categorized according to the timing of the first surfactant dose as no surfactant, first administration within the first hour of life, or first administration after the first hour. This mutually exclusive timing-based classification was reported in Table 1 and used in the expanded propensity score and early respiratory severity sensitivity analyses.

Because gestational age and birth weight are strongly related measures of maturity, they were evaluated in separate multivariable and propensity score models.

### 2.5. Multivariable and Propensity Score Analyses

For the conventional multivariable analyses, separate gestational age-based and birth weight-based logistic regression models were constructed for BPD and BPD or death. The extended gestational age-based model included caffeine initiation timing, gestational age, sex, antenatal corticosteroid therapy, Apgar score at 5 min, pretreatment FiO requirement, and early surfactant treatment. The extended birth weight-based model included birth weight instead of gestational age, with the same remaining covariates.

Expanded propensity score models were subsequently constructed to address maternal, placental, perinatal, and early respiratory confounding. The propensity score represented the probability of caffeine initiation after the first 2 h conditional on measured covariates.

The expanded gestational age-based propensity score model included gestational age, sex, antenatal corticosteroid therapy, Apgar score at 5 min, gestational diabetes mellitus, premature or preterm premature rupture of membranes, preeclampsia, placental abruption, cervical insufficiency, multiple gestation, Fenton-based small-for-gestational-age status, highest pretreatment FiO_2_ requirement, any pretreatment respiratory support, invasive mechanical ventilation during the first hour, and surfactant administration within the first hour. The expanded birth weight-based model included birth weight instead of gestational age, with the same remaining covariates.

Stabilized inverse probability of treatment weights were calculated from the expanded propensity scores. Covariate balance before and after weighting was evaluated using absolute standardized mean differences. An absolute standardized mean difference <0.10 was considered indicative of adequate balance. Weight distributions, maximum weights, and effective sample sizes were also examined to identify possible positivity violations or excessive influence from extreme weights.

Weighted generalized linear models with a binomial distribution, logit link, and robust covariance estimation were used to evaluate BPD, BPD or death, and moderate/severe BPD or death. Because a small residual imbalance remained for any pretreatment respiratory support, an additional covariate-adjusted weighted sensitivity analysis was performed for BPD and BPD or death.

Additional parsimonious logistic regression sensitivity models were constructed for BPD and BPD or death. These models included caffeine initiation timing, either gestational age or birth weight, invasive mechanical ventilation during the first hour, and surfactant administration within the first hour. Additional multivariable models were not constructed for death alone or moderate/severe BPD or death because of the limited number of events and the associated risk of separation and unstable estimation.

### 2.6. Statistical Analysis

Statistical analyses were performed using IBM SPSS Statistics for Windows, version 25.0 (IBM Corp., Armonk, NY, USA). Continuous variables were assessed for normality using the Shapiro–Wilk test. Normally distributed variables are presented as mean ± standard deviation and were compared using Student’s *t*-test. Non-normally distributed variables are presented as median and interquartile range and were compared using the Mann–Whitney U test. Categorical variables are presented as number and percentage and were compared using the chi-square test, Fisher’s exact test, or Monte Carlo exact test, as appropriate.

The main exposure was caffeine citrate initiation after the first 2 h of life, with initiation within the first 2 h as the reference category. Odds ratios and 95% confidence intervals were calculated.

No missing-data imputation was performed. Analyses were conducted using available-case or complete-case methods, depending on the variables required for each model. Denominators are therefore reported for outcomes with missing or non-assessable data. Three infants who died before 36 weeks’ PMA were excluded from the survivor-based BPD outcome but remained in the composite outcomes.

A sensitivity power analysis for the unadjusted comparison of two independent proportions was performed based on the available group sizes of 41 and 43 infants. With a two-sided α level of 0.05 and 80% power, the minimum detectable standardized effect size was approximately Cohen’s h = 0.61, indicating that the available sample could detect only relatively large between-group differences. A two-sided *p* value < 0.05 was considered statistically significant.

## 3. Results

### 3.1. Study Population

During the study period, 379 infants were admitted to the neonatal intensive care unit. After exclusion of 295 infants, 84 infants were included in the study cohort (Figure 1). Caffeine citrate was initiated within the first 2 h of life in 41 infants and after the first 2 h, but within the first 24 h, in 43 infants. BPD could be assessed in 81 infants; three infants died before assessment at 36 weeks’ PMA and were included only in the composite outcomes.

Among infants born at <32 weeks’ gestation with a birth weight of ≤1500 g who were assessable for BPD at 36 weeks’ PMA, BPD occurred in 63 of 150 infants (42%).

Four infants had missing Apgar scores at 5 min. Consequently, the expanded propensity score analyses included 80 infants. Among these, BPD was assessable in 77 infants, whereas BPD or death and moderate/severe BPD or death were assessable in all 80 infants.

### 3.2. Baseline Characteristics

Baseline characteristics are presented in Table 1. Gestational age and birth weight were numerically lower in infants who received caffeine after the first 2 h, although the between-group differences were not statistically significant. Maternal age was higher and the Apgar score at 1 min was lower in the after 2 h group.

The groups did not differ significantly in sex, multiple gestation, mode of delivery, gestational diabetes mellitus, preeclampsia, premature or preterm premature rupture of membranes, placental abruption, cervical insufficiency, Fenton-based small-for-gestational-age status, antenatal corticosteroid therapy, Apgar score at 5 min, SNAPPE-II score, pretreatment FiO_2_ requirement, pretreatment respiratory support category, first-hour invasive mechanical ventilation, or overall surfactant timing.

### 3.3. Respiratory and Neonatal Outcomes

Respiratory outcomes, neonatal morbidities, caffeine treatment characteristics, and length of neonatal intensive care unit stay are presented in Table 2.

Among infants assessable for BPD, BPD occurred in 24/40 (60.0%) infants in the after 2 h group and 10/41 (24.4%) in the within 2 h group (OR 4.65, 95% CI 1.79–12.06; *p* = 0.002). BPD or death occurred in 27/43 (62.8%) and 10/41 (24.4%), respectively (OR 5.23, 95% CI 2.04–13.44; *p* < 0.001).

Moderate/severe BPD and moderate/severe BPD or death were also more frequent among infants who received caffeine after the first 2 h. Supplemental oxygen and respiratory support requirements at several postnatal time points were generally more frequent in the after 2 h group. Respiratory support at 36 weeks’ PMA was numerically more frequent in this group, although the comparison did not reach conventional statistical significance.

The median neonatal intensive care unit length of stay was 52 days in the within 2 h group and 63 days in the after 2 h group; this difference was not statistically significant.

Among non-respiratory neonatal outcomes, pulmonary interstitial emphysema was more frequent in the after 2 h group. Severe intraventricular hemorrhage and death were numerically more frequent after later caffeine initiation, but the differences were not statistically significant. No consistent group differences were observed for necrotizing enterocolitis stage ≥ II, retinopathy of prematurity stage 3–4, or patent ductus arteriosus treatment.

### 3.4. Multivariable Logistic Regression Analyses

The conventional multivariable logistic regression models are presented in Table 3. Caffeine initiation after the first 2 h remained associated with BPD and BPD or death in both the extended gestational age-based and birth weight-based models.

In the gestational age-based model, the OR was 10.76 (95% CI 1.73–66.98; *p* = 0.011) for BPD and 13.49 (95% CI 2.19–83.32; *p* = 0.005) for BPD or death. In the birth weight-based model, the corresponding ORs were 18.94 (95% CI 2.01–178.65; *p* = 0.010) and 23.35 (95% CI 2.52–216.19; *p* = 0.006). The wide confidence intervals indicate substantial uncertainty regarding the magnitude of these associations.

### 3.5. Expanded Propensity Score and IPTW Analyses

Results of the expanded IPTW analyses are presented in Table 4. Both propensity score models included 80 complete cases.

The stabilized weights were well behaved. Mean weights were approximately 0.98 in both models. The maximum stabilized weight was 1.772 in the gestational age-based model and 2.002 in the birth weight-based model. The corresponding effective sample sizes were 73.07 and 72.26, respectively.

**Figure 2:**
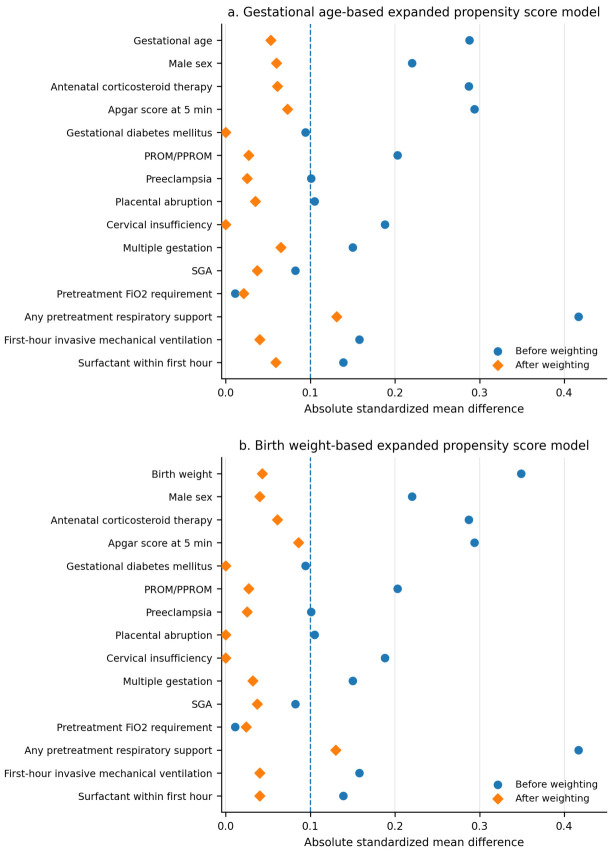
Covariate balance before and after expanded stabilized inverse probability of treatment weighting. Absolute standardized mean differences are shown before and after weighting. The vertical dashed line indicates the 0.10 threshold for acceptable covariate balance. (**a**) Gestational age-based propensity score model. (**b**) Birth weight-based propensity score model.

Weighting substantially improved covariate balance (Figure 2 and Table S1). All measured covariates achieved an absolute standardized mean difference below 0.10 after weighting except for a small residual imbalance in any pretreatment respiratory support. The post-weighting absolute standardized mean difference for this variable was 0.131 in the gestational age-based model and 0.130 in the birth weight-based model.

In the expanded gestational age-based IPTW analysis, caffeine initiation after the first 2 h was associated with higher odds of BPD (OR 3.40, 95% CI 1.22–9.48; *p* = 0.019) and BPD or death (OR 3.89, 95% CI 1.41–10.71; *p* = 0.009). In the expanded birth weight-based analysis, the corresponding ORs were 3.23 (95% CI 1.16–9.00; *p* = 0.025) and 3.68 (95% CI 1.34–10.12; *p* = 0.012).

Later caffeine initiation was also associated with moderate/severe BPD or death in the gestational age-based IPTW model (OR 9.92, 95% CI 1.18–83.48; *p* = 0.035) and the birth weight-based model (OR 9.53, 95% CI 1.13–80.27; *p* = 0.038). These estimates were based on only 11 composite events and had very wide confidence intervals.

Additional adjustment for the small residual imbalance in any pretreatment respiratory support produced similar estimates. The adjusted weighted ORs for BPD were 3.34 (95% CI 1.19–9.33; *p* = 0.022) and 3.17 (95% CI 1.14–8.86; *p* = 0.028) in the gestational age- and birth weight-based models, respectively. Corresponding ORs for BPD or death were 3.80 (95% CI 1.38–10.52; *p* = 0.010) and 3.60 (95% CI 1.30–9.96; *p* = 0.014).

A corresponding covariate-adjusted weighted model for moderate/severe BPD or death could not be reliably estimated because quasi-complete separation occurred.

### 3.6. Early Respiratory Severity Sensitivity Analyses

The association between first-hour invasive mechanical ventilation and surfactant administration within the first hour was strong (phi = 0.690; *p* < 0.001). Nevertheless, there was not complete overlap between the two variables, and models including both variables converged without separation for BPD and BPD or death.

In additional logistic regression analyses adjusting for either gestational age or birth weight together with first-hour invasive mechanical ventilation and surfactant administration within the first hour, caffeine initiation after the first 2 h remained associated with both BPD and BPD or death (Table S2).

For BPD, the OR was 15.82 (95% CI 2.93–85.41; *p* = 0.001) in the gestational age-based model and 23.61 (95% CI 2.88–193.56; *p* = 0.003) in the birth weight-based model. For BPD or death, the corresponding ORs were 17.39 (95% CI 3.20–94.61; *p* = 0.001) and 26.15 (95% CI 3.15–216.97; *p* = 0.002).

These models support the persistence of the association after adjustment for measured golden-hour respiratory interventions, but their wide confidence intervals preclude precise interpretation of the effect magnitude.

## 4. Discussion

In this single-center observational cohort of very preterm infants, caffeine citrate initiation after the first 2 h of life was associated with higher frequencies of BPD, BPD or death, and moderate/severe BPD or death. The association persisted across conventional multivariable models and expanded IPTW analyses incorporating maternal, placental, perinatal, and golden-hour respiratory variables. In the expanded IPTW models, later initiation was associated with approximately three- to four-fold higher odds of BPD and BPD or death. Additional adjustment for the small residual imbalance in pretreatment respiratory support produced similar estimates.

These findings are consistent with previous evidence suggesting that earlier caffeine treatment may be associated with more favorable respiratory outcomes in preterm infants. The CAP trial demonstrated that caffeine treatment reduced BPD and improved survival without neurodevelopmental disability [3,4]. Subsequent observational studies and meta-analyses have associated earlier caffeine initiation with shorter respiratory support, reduced exposure to invasive ventilation, and lower BPD risk [5,6,7,8]. However, most previous studies defined early treatment as caffeine administration within the first 24–72 h, whereas the present study examined a substantially narrower interval during immediate postnatal care.

The 2 h threshold used in this study requires careful interpretation. It was selected because the unit protocol targeted caffeine loading within the first 2 h and not because 2 h represents an established biological or pharmacodynamic boundary. Accordingly, the present findings do not demonstrate that a discrete physiological change occurs at 2 h. Rather, they evaluate whether completion of caffeine administration within a protocol-defined early-care window is associated with subsequent respiratory outcomes.

Confounding by early illness severity is a central concern. Infants requiring intubation, surfactant administration, vascular access, and other urgent procedures may receive caffeine later and may also have a greater underlying risk of BPD or death. Maternal and placental complications, including placental abruption, may additionally influence neonatal respiratory and survival outcomes [13].

To address these concerns, the expanded propensity score models incorporated gestational diabetes, premature or preterm premature rupture of membranes, preeclampsia, placental abruption, cervical insufficiency, multiple gestation, small-for-gestational-age status, highest pretreatment FiO_2_, any pretreatment respiratory support, first-hour invasive mechanical ventilation, and surfactant administration within the first hour, in addition to maturity and other baseline characteristics. Weight distributions and effective sample sizes were acceptable, and covariate balance improved substantially.

A small residual imbalance remained for any pretreatment respiratory support. However, adding this variable to the weighted outcome models produced little change in the estimates for BPD and the composite outcome of BPD or death. Furthermore, the association persisted in separate models that explicitly adjusted for first-hour invasive mechanical ventilation and surfactant administration within the first hour. These analyses strengthen the consistency of the finding across measured confounders but cannot exclude residual confounding from unrecorded features such as oxygen saturation trajectories, early blood gas abnormalities, detailed respiratory severity scores, or the sequence and duration of delivery-room interventions.

The BPD or death outcome is particularly important because three infants who died before 36 weeks’ PMA could not be included in the survivor-based BPD analysis. Excluding such infants from all analyses could introduce survivor-related bias. The composite outcome retained these infants and produced findings similar to those of the BPD analysis. A formal competing-risk or hierarchical analysis was not considered reliable because only six deaths occurred.

The association with moderate/severe BPD or death should be interpreted especially cautiously. Only 11 infants experienced this composite outcome, and the resulting confidence intervals were very wide. Additional covariate adjustment produced quasi-complete separation, demonstrating that the data did not support more complex modeling of this sparse outcome. The corresponding results should therefore be considered exploratory rather than a precise estimate of the magnitude of association.

The conventional sensitivity models produced larger conditional ORs than the IPTW models. This difference should not be interpreted as evidence that caffeine delay increases risk by a specific 16- to 26-fold magnitude. Conditional and weighted marginal odds ratios estimate different quantities, and odds ratios are non-collapsible. In addition, the small sample and strong associations between maturity, respiratory severity, and the outcomes contributed to unstable estimates. The consistency in direction and statistical significance is more informative than the exact magnitude of these large estimates.

The modest sample size represents an important limitation of this study. Because all eligible infants treated during the prespecified study period were included, no a priori sample size calculation was performed. The sensitivity power analysis indicated that the available sample was capable of detecting only relatively large between-group differences; smaller but potentially clinically relevant associations may therefore have remained undetected. In addition, the small number of events for some secondary outcomes resulted in wide confidence intervals and limited the precision of the corresponding estimates. Therefore, findings related to infrequent secondary outcomes should be interpreted as exploratory.

Other limitations include the single-center design and the non-randomized determination of caffeine timing. Unit-specific protocols, staffing, availability of vascular access, and the sequencing of stabilization procedures may affect whether caffeine can be administered within 2 h. These factors limit generalizability to units with different workflows. The indication and urgency category for cesarean delivery were not available, preventing adjustment for category-1 emergency cesarean delivery. Detailed oxygen saturation trends and validated first-hour respiratory severity scores were also unavailable.

Caffeine discontinuation and treatment duration were determined during the subsequent clinical course. These variables may be influenced by respiratory recovery and may function as consequences or mediators of earlier clinical status rather than baseline confounders. Differences in oxygen requirements at caffeine discontinuation and one week later were therefore considered descriptive and were not included in models examining the association between initiation timing and BPD-related outcomes.

Non-respiratory outcomes showed no consistent association with caffeine timing. Pulmonary interstitial emphysema was more frequent in the later group, whereas differences in severe intraventricular hemorrhage and death did not reach statistical significance. These comparisons were based on limited event numbers and should not be interpreted as evidence of independent effects.

The study also has strengths. It focused on a narrowly defined and clinically relevant early-care window, included detailed caffeine timing, evaluated deaths through composite outcomes, and used complementary conventional and propensity score-based approaches. The expanded propensity score models incorporated maternal, placental, perinatal, and golden-hour respiratory characteristics. Balance diagnostics, effective sample sizes, and maximum weights were transparently reported. Nevertheless, statistical adjustment cannot substitute for randomization, and the results establish association rather than causation.

Larger prospective multicenter studies are needed to determine whether protocolized caffeine administration during the first postnatal hours independently improves respiratory outcomes or whether early administration primarily reflects broader differences in stabilization efficiency and illness severity.

## 5. Conclusions

Caffeine citrate initiation after the first 2 h of life was associated with higher odds of BPD and BPD or death in very preterm infants. The association persisted after expanded stabilized IPTW analyses incorporating maternal, placental, perinatal, and golden-hour respiratory variables and after additional adjustment for first-hour invasive mechanical ventilation and surfactant administration within the first hour.

Because of the observational design, modest sample size, wide confidence intervals, and potential residual confounding, these findings should not be interpreted as evidence of a causal effect or as a precise estimate of the magnitude of benefit associated with caffeine administration within 2 h. Prospective multicenter studies are required to determine whether protocolized very early caffeine initiation independently improves respiratory outcomes.

## Figures and Tables

**Table 1:** Baseline characteristics according to caffeine citrate initiation timing.

Variable	Within 2 h Initiation (n = 41)	After 2 h Initiation (n = 43)	*p* Value
Gestational age, weeks	28.4 ± 2.3	27.7 ± 2.0	0.156
Birth weight, g	1100 (900–1375)	980 (795–1299)	0.088
Fenton-based SGA	2/41 (4.9)	3/43 (7.0)	1.000
Maternal age, years	25 (24–32)	30 (26–33.5)	0.044
Male sex	15/41 (36.6)	21/43 (48.8)	0.257
Multiple gestation	4/41 (9.8)	6/43 (14.0)	0.739
Cesarean delivery	40/41 (97.6)	41/43 (95.3)	1.000
Gestational diabetes mellitus	5/41 (12.2)	4/43 (9.3)	0.735
Preeclampsia	8/41 (19.5)	7/43 (16.3)	0.780
PROM/PPROM	9/41 (22.0)	7/43 (16.3)	0.585
Placental abruption	4/41 (9.8)	3/43 (7.0)	0.710
Cervical insufficiency	4/41 (9.8)	2/43 (4.7)	0.427
Antenatal corticosteroid therapy	19/41 (46.3)	13/43 (30.2)	0.129
Apgar score at 1 min	5 (3.2–6)	3 (2–6)	0.036
Apgar score at 5 min	7 (6–8)	6 (5–8)	0.134
Apgar score at 10 min	7 (6–8)	7 (6.5–8)	0.630
SNAPPE-II score	60 (29–66)	45.5 (22.5–59.5)	0.197
Pretreatment FiO_2_ requirement, %	30 (25–40)	35 (30–40)	0.433
Pretreatment respiratory support type			
Absent	5/41 (12.2)	1/43 (2.3)	0.273
Incubator oxygen	1/41 (2.4)	1/43 (2.3)	
nCPAP	12/41 (29.3)	9/43 (20.9)	
NSIMV	4/41 (9.8)	8/43 (18.6)	
Invasive mechanical ventilation	19/41 (46.3)	24/43 (55.8)	
First-hour invasive mechanical ventilation	19/41 (46.3)	24/43 (55.8)	0.513
Surfactant timing			
No surfactant	18/41 (43.9)	12/43 (27.9)	0.210
Within the first hour	20/41 (48.8)	24/43 (55.8)	
After the first hour	3/41 (7.3)	7/43 (16.3)	
Total surfactant dose requirement	1 (0–1)	1 (0–1)	0.054

Note: Values are presented as mean ± standard deviation, median (interquartile range), or n/N (%), as appropriate. Pretreatment respiratory support type refers to the highest level of respiratory support required before caffeine citrate initiation. Surfactant timing was categorized according to the timing of the first surfactant dose as no surfactant, administration within the first hour of life, or administration after the first hour of life. For multi-category variables, *p* values were calculated using Fisher’s exact test or Monte Carlo exact test, as appropriate. Abbreviations: FiO_2_, fraction of inspired oxygen; nCPAP, nasal continuous positive airway pressure; NSIMV, nasal synchronized intermittent mandatory ventilation; PROM, premature rupture of membranes; PPROM, preterm premature rupture of membranes; SGA, small for gestational age; SNAPPE-II, Score for Neonatal Acute Physiology with Perinatal Extension-II.

**Table 2:** Caffeine treatment course, respiratory outcomes, and neonatal morbidities according to caffeine citrate initiation timing.

Variable	Within 2 h Initiation	After 2 h Initiation	*p* Value
Caffeine treatment course			
Total duration of caffeine citrate therapy, days	37 (28–56)	45 (28.5–59)	0.447
PMA on last day of caffeine citrate dosing, weeks	34.4 (33–36)	34 (32.9–35.2)	0.148
Caffeine discontinuation ≥ 34 weeks’ PMA	26/41 (63.4)	22/43 (51.2)	0.257
Duration between last caffeine dose and discharge, days	9.5 (7–20.2)	17.5 (10–24)	0.043
FiO_2_ at caffeine discontinuation, %	21 (21–21)	21 (21–21)	0.037
FiO_2_ 1 week after caffeine discontinuation, %	21 (21–21)	21 (21–21)	0.045
PMA at hospital discharge or death, weeks	36 (35–37.3)	36 (35–38.2)	0.332
Length of NICU stay until discharge or death, days	52 (35–72)	63 (44–80)	0.130
Respiratory outcomes			
Supplemental oxygen requirement at postnatal day 28	12/41 (29.3)	26/42 (61.9)	0.003
Supplemental oxygen requirement at PMA 32 weeks	13/41 (31.7)	18/40 (45.0)	0.218
Supplemental oxygen requirement at PMA 34 weeks	9/41 (22.0)	14/39 (35.9)	0.168
Supplemental oxygen requirement at PMA 36 weeks	3/41 (7.3)	6/38 (15.8)	0.300
Respiratory support at postnatal day 28	15/41 (36.6)	26/42 (61.9)	0.021
Respiratory support at PMA 32 weeks	15/41 (36.6)	20/40 (50.0)	0.223
Respiratory support at PMA 34 weeks	9/41 (22.0)	15/40 (37.5)	0.125
Respiratory support at PMA 36 weeks	3/41 (7.3)	9/38 (23.7)	0.060
BPD among evaluable infants	10/41 (24.4)	24/40 (60.0)	0.001
BPD or death	10/41 (24.4)	27/43 (62.8)	<0.001
Moderate/severe BPD	1/41 (2.4)	7/40 (17.5)	0.029
Moderate/severe BPD or death	1/41 (2.4)	10/43 (25.6)	0.007
BPD severity distribution			
No BPD	31/41 (75.6)	16/40 (40.0)	0.007
Mild BPD	9/41 (22.0)	17/40 (42.5)	
Moderate BPD	1/41 (2.4)	3/40 (7.5)	
Severe BPD	0/41 (0.0)	4/40 (10.0)	
Neonatal morbidities			
Hydrocortisone treatment	9/41 (22.0)	4/42 (9.5)	0.141
Any PDA treatment	9/41 (22.0)	14/43 (32.6)	0.276
Surgical PDA treatment	0/41 (0.0)	1/43 (2.3)	1.000
Pulmonary interstitial emphysema	0/41 (0.0)	9/43 (20.9)	0.002
Early-onset neonatal sepsis	34/41 (82.9)	28/43 (65.1)	0.063
Late-onset neonatal sepsis	5/41 (12.2)	10/41 (24.4)	0.153
NEC stage ≥II	2/41 (4.9)	3/43 (7.0)	1.000
ROP stage 3–4	1/41 (2.4)	3/43 (7.0)	0.616
Any IVH	3/41 (7.3)	7/43 (16.3)	0.314
IVH grade 3–4	1/41 (2.4)	6/43 (14.0)	0.110
Death	1/41 (2.4)	5/43 (11.6)	0.202

Note: Values are presented as median (interquartile range) or n/N (%), as appropriate. Supplemental oxygen requirement was defined as FiO_2_ > 21% at the corresponding time point. The *p* value for BPD severity distribution represents the overall comparison across BPD severity categories. PMA and NICU stay were calculated at hospital discharge for survivors and at death for infants who died before discharge. Moderate/severe BPD or death was analyzed as a composite outcome, and infants who both developed moderate/severe BPD and died were counted only once. Abbreviations: BPD, bronchopulmonary dysplasia; FiO_2_, fraction of inspired oxygen; IVH, intraventricular hemorrhage; NEC, necrotizing enterocolitis; NICU, neonatal intensive care unit; PDA, patent ductus arteriosus; PMA, postmenstrual age; ROP, retinopathy of prematurity.

**Table 3:** Multivariable logistic regression models for BPD and BPD or death.

Outcome	Model	OR for After 2 h Initiation	95% CI	*p* Value
BPD	Unadjusted	4.65	1.79–12.06	0.002
BPD	Extended GA-based model	10.76	1.73–66.98	0.011
BPD	Extended BW-based model	18.94	2.01–178.65	0.010
BPD or death	Unadjusted	5.23	2.04–13.44	<0.001
BPD or death	Extended GA-based model	13.49	2.19–83.32	0.005
BPD or death	Extended BW-based model	23.35	2.52–216.19	0.006

Note: Odds ratios indicate the odds of the outcome in infants who received caffeine citrate after the first 2 h of life compared with those who received caffeine citrate within the first 2 h. The extended GA-based model included caffeine initiation timing, gestational age, sex, antenatal corticosteroid therapy, Apgar score at 5 min, pretreatment FiO_2_ requirement, and early surfactant therapy. The extended BW-based model included birth weight instead of gestational age, with the same remaining covariates. Abbreviations: BPD, bronchopulmonary dysplasia; BW, birth weight; CI, confidence interval; GA, gestational age; OR, odds ratio.

**Table 4:** Expanded stabilized IPTW analyses for BPD-related outcomes.

Outcome	Analysis	OR for After 2 h Initiation	95% CI	*p* Value
BPD	Expanded GA-based IPTW	3.40	1.22–9.48	0.019
BPD	Expanded BW-based IPTW	3.23	1.16–9.00	0.025
BPD or death	Expanded GA-based IPTW	3.89	1.41–10.71	0.009
BPD or death	Expanded BW-based IPTW	3.68	1.34–10.12	0.012
Moderate/severe BPD or death	Expanded GA-based IPTW	9.92	1.18–83.48	0.035
Moderate/severe BPD or death	Expanded BW-based IPTW	9.53	1.13–80.27	0.038

Note: Odds ratios indicate the odds of the outcome in infants who received caffeine citrate after the first 2 h of life compared with those who received caffeine citrate within the first 2 h. The expanded GA-based propensity score model included gestational age, sex, antenatal corticosteroid therapy, Apgar score at 5 min, gestational diabetes mellitus, PROM/PPROM, preeclampsia, placental abruption, cervical insufficiency, multiple gestation, SGA, pretreatment FiO_2_ requirement, any pretreatment respiratory support, first-hour invasive mechanical ventilation, and surfactant administration within the first hour. The expanded BW-based model included birth weight instead of gestational age, with the same remaining covariates. Stabilized IPTW analyses were performed using weighted generalized linear models with robust covariance estimation. Effective sample sizes were 73.07 and 72.26, and maximum stabilized weights were 1.77 and 2.00, in the GA- and BW-based models, respectively. BPD analyses included 77 infants; composite outcome analyses included 80 infants. Abbreviations: BPD, bronchopulmonary dysplasia; BW, birth weight; CI, confidence interval; GA, gestational age; IPTW, inverse probability of treatment weighting; OR, odds ratio.

## Data Availability

The data presented in this study are available on reasonable request from the corresponding author. The data are not publicly available due to ethical and privacy restrictions related to clinical data of premature infants.

## Supplementary Materials

The following supporting information can be downloaded at: https://www.mdpi.com/article/10.3390/children13070968/s1, Table S1: Covariate balance before and after expanded stabilized inverse probability of treatment weighting. Table S2: Early respiratory severity-adjusted logistic regression models for BPD and BPD or death.

## Abbreviations

The following abbreviations are used in this manuscript:

BPD	Bronchopulmonary dysplasia
BW	Birth weight
CI	Confidence interval
FiO2	Fraction of inspired oxygen
GA	Gestational age
IPTW	Inverse probability of treatment weighting
IVH	Intraventricular hemorrhage
NEC	Necrotizing enterocolitis
NICU	Neonatal intensive care unit
OR	Odds ratio
PDA	Patent ductus arteriosus
PMA	Postmenstrual age
PPROM	Preterm premature rupture of membranes
PROM	Premature rupture of membranes
PS	Propensity score
ROP	Retinopathy of prematurity
SGA	Small for gestational age
SMD	Standardized mean difference
SNAPPE-II	Score for Neonatal Acute Physiology with Perinatal Extension-II
