# Supplementary material for: Association Between Caffeine Citrate Initiation Within the First 2 h of Life and Respiratory Outcomes in Very Preterm Infants: A Single-Center Observational Cohort Study
Source: Children (Basel). 2026 Jul 22;13(7):968. doi: 10.3390/children13070968 (PMC13407232; doi:10.3390/children13070968)
Supplement: Supplementary file 1 [file children-13-00968-s001.zip › children-4416143-supplementary.pdf]

Table S1: Covariate balance before and after expanded stabilized inverse probability of treatment weighting

Covariate	Absolute SMD before IPTW	Absolute SMD after IPTW
**GA-based propensity score model**		
Gestational age	0.288	0.053
Male sex	0.220	0.060
Antenatal corticosteroid therapy	0.287	0.061
Apgar score at 5 min	0.294	0.073
Gestational diabetes mellitus	0.094	0.000
PROM/PPROM	0.203	0.027
Preeclampsia	0.101	0.025
Placental abruption	0.105	0.035
Cervical insufficiency	0.188	0.000
Multiple gestation	0.150	0.065
SGA	0.082	0.037
Pretreatment FiO2 requirement	0.011	0.021
Any pretreatment respiratory support	0.417	0.131
First-hour invasive mechanical ventilation	0.158	0.040
Surfactant within first hour	0.139	0.059
**BW-based propensity score model**		
Birth weight	0.349	0.043
Male sex	0.220	0.040
Antenatal corticosteroid therapy	0.287	0.061
Apgar score at 5 min	0.294	0.086
Gestational diabetes mellitus	0.094	0.000
PROM/PPROM	0.203	0.027
Preeclampsia	0.101	0.025
Placental abruption	0.105	0.000
Cervical insufficiency	0.188	0.000
Multiple gestation	0.150	0.032
SGA	0.082	0.037
Pretreatment FiO2 requirement	0.011	0.024
Any pretreatment respiratory support	0.417	0.130
First-hour invasive mechanical ventilation	0.158	0.040
Surfactant within first hour	0.139	0.040

Note: Absolute standardized mean differences <0.10 were considered indicative of adequate covariate balance. Expanded stabilized IPTW substantially improved balance in both models. A small residual imbalance remained for any pretreatment respiratory support (absolute SMD, 0.131 in the GA-based model and 0.130 in the BW-based model). The complete-case propensity score cohorts included 80 infants. Effective sample sizes were 73.07 and 72.26, and maximum stabilized weights were 1.77 and 2.00, in the GA- and BW-based models, respectively.

Abbreviations: BW, birth weight; FiO2, fraction of inspired oxygen; GA, gestational age; IPTW, inverse probability of treatment weighting; PROM, premature rupture of membranes; PPROM, preterm premature rupture of membranes; SGA, small for gestational age; SMD, standardized mean difference.

Table S2. Early respiratory severity-adjusted logistic regression models for BPD and BPD or death

Outcome	Model	Predictor	Adjusted OR	95% CI	p value
**BPD**	**GA-based**	**Caffeine initiation after first 2 hours**	**15.824**	**2.932–85.412**	**0.001**
		Gestational age, per 1-week increase	0.433	0.259–0.724	0.001
		First-hour invasive mechanical ventilation	1.653	0.326–8.370	0.544
		Surfactant within first hour	6.914	1.155–41.394	0.034
**BPD**	**BW-based**	**Caffeine initiation after first 2 hours**	**23.612**	**2.880–193.556**	**0.003**
		Birth weight, per 100-g increase	0.461	0.302–0.706	<0.001
		First-hour invasive mechanical ventilation	1.000	0.174–5.744	1.000
		Surfactant within first hour	7.142	0.877–58.182	0.066
**BPD or death**	**GA-based**	**Caffeine initiation after first 2 hours**	**17.386**	**3.195–94.611**	**0.001**
		Gestational age, per 1-week increase	0.414	0.247–0.694	0.001
		First-hour invasive mechanical ventilation	1.545	0.305–7.836	0.600
		Surfactant within first hour	6.634	1.105–39.809	0.038
**BPD or death**	**BW-based**	**Caffeine initiation after first 2 hours**	**26.153**	**3.152–216.968**	**0.002**
		Birth weight, per 100-g increase	0.449	0.294–0.685	<0.001
		First-hour invasive mechanical ventilation	0.949	0.165–5.464	0.953
		Surfactant within first hour	7.052	0.852–58.358	0.070

Note: Adjusted odds ratios were estimated using parsimonious logistic regression models specified for this sensitivity analysis. GA-based models included caffeine initiation timing, gestational age, first-hour invasive mechanical ventilation, and surfactant administration within the first hour; BW-based models replaced gestational age with birth weight per 100-g increase. The BPD models included 81 infants and the BPD or death models included 84 infants. Hosmer–Lemeshow p values were 0.888, 0.779, 0.880, and 0.654 for the BPD GA-based, BPD BW-based, BPD or death GA-based, and BPD or death BW-based models, respectively. First-hour invasive mechanical ventilation and surfactant administration within the first hour were strongly associated (phi=0.690, p<0.001); their individual coefficients should therefore be interpreted cautiously. Models for moderate/severe BPD or death and death were not fitted because of sparse events and separation risk.

Abbreviations: BPD, bronchopulmonary dysplasia; BW, birth weight; CI, confidence interval; GA, gestational age; OR, odds ratio.
